# Cervical cancer research disparities among African immigrant women in the United States: A systematic review

**DOI:** 10.1017/S1478951526101849

**Published:** 2026-02-24

**Authors:** Dukanwojo Beulah Suleman, Sunkanmi Folorunsho

**Affiliations:** Department of Sociology, University of Nebraska-Lincoln, Nebraska, USA

**Keywords:** African immigrant women, cervical cancer screening, HPV vaccination, health disparities, United States

## Abstract

**Background:**

Previous studies indicate that African immigrant women in the United States have lower rates of cervical cancer screening and prevention than other racial and immigrant groups, with additional heterogeneity by country of origin, language proficiency, and length of U.S. residence.

**Objectives:**

This review aimed to (a) summarize barriers and facilitators to screening, (b) examine how existing studies conceptualize African immigrant identity and employ disaggregated analyses, and (c) apply intersectionality and stress process frameworks to highlight structural determinants shaping screening behaviors.

**Methods:**

This systematic review, registered with PROSPERO (CRD420251151600), synthesizes evidence on cervical cancer screening and HPV vaccination among African immigrant women in the United States. PubMed, ProQuest, EBSCO, PsycINFO, MEDLINE, Scopus, and Google Scholar were searched for peer-reviewed studies published between January 2010 and December 2024. Seventeen studies met inclusion criteria, including cross-sectional surveys (n = 7), qualitative studies (n = 5), mixed-methods studies (n = 3), retrospective cohort analyses (n = 1), and one randomized controlled trial.

**Results:**

Only 11 of the 17 studies disaggregated African immigrant women by country of origin or related subgroup characteristics. Risk of bias was assessed using the Newcastle–Ottawa Scale for observational studies and the Critical Appraisal Skills Programme checklist for qualitative studies. Across studies, African immigrant women consistently faced barriers to screening, including language discordance, lack of insurance, limited HPV awareness, cultural stigma, and unfamiliarity with the U.S. healthcare system. Interventions such as HPV self-sampling and culturally tailored education showed promise in improving screening uptake.

**Significance of Results:**

The findings point to the need for standardized disaggregated data collection, culturally responsive interventions, and theory-driven research to reduce cervical cancer prevention disparities among African immigrant women in the United States.

## Introduction

Although cervical cancer-related mortality has declined substantially following the widespread implementation of cytological screening programs, HPV testing, and effective HPV vaccines, cervical cancer remains a significant public health concern in the United States due to persistent disparities in screening utilization across racial and immigrant groups (American Cancer Society [ACS], 2025). Recent U.S. cancer surveillance data indicate that non-Hispanic Black women experience nearly 70% higher cervical cancer mortality compared to non-Hispanic White women, while Hispanic women face approximately 40% higher incidence rates (CDC, 2025). These patterns underline the continued influence of structural and social determinants, including socioeconomic status, healthcare access, and systemic racism, on cervical cancer outcomes despite overall national progress (Warnecke et al. 2008; Benard et al. 2014).

African immigrant women represent one of the fastest-growing immigrant populations in the United States, with population growth exceeding 90% between 2000 and 2020 (Anderson 2022). Many originate from sub-Saharan Africa, a region that bears the highest global burden of cervical cancer due to limited screening infrastructure, late-stage diagnosis, and constrained access to treatment (Ferlay et al. 2013; World Health Organization 2020). Following migration, African immigrant women frequently encounter intersecting barriers to preventive care, including language discordance, lack of insurance, cultural stigma surrounding gynecological examinations, and unfamiliarity with U.S. healthcare systems (Ndukwe et al. 2013; Omenka et al. 2020). Despite these vulnerabilities, African immigrant women remain underrepresented in U.S. cervical cancer research and health surveillance, as many studies aggregate them within broad “Black” or “foreign-born” categories (Castañeda et al. 2015). Such aggregation obscures heterogeneity by country of origin, migration history, language, and cultural context, contributing to what has been described as “data invisibility” in health equity research (Ponce et al. 2025).

Existing evidence suggests that immigrant women, particularly those from African countries, experience disproportionately low rates of cervical cancer screening due to limited HPV knowledge, fear of diagnosis, stigma surrounding reproductive health, and structural barriers such as transportation challenges and healthcare costs (Akinlotan et al. 2017; Okoronkwo et al. 2021). Language barriers further compound these disparities, as limited English proficiency is consistently associated with reduced screening adherence and lower health literacy (Jacobs et al. 2006; Sentell et al. 2015). However, the evidence base remains fragmented, with relatively few studies disaggregating African immigrant subgroups or explicitly examining how race, gender, and immigration intersect to shape screening behaviors.

Two complementary theoretical frameworks provide critical insight into these patterns. Intersectionality theory highlights how overlapping systems of racism, sexism, and nativism create distinct constraints for African immigrant women, whose experiences do not fully align with those of U.S.-born Black women or other immigrant groups (Crenshaw 1991). The stress process model further emphasizes how migration-related stressors, discrimination, and resource scarcity influence health behaviors through pathways involving coping resources, social support, and psychological well-being (Pearlin et al. 1981). In essence, these perspectives point to the importance of disaggregated data and culturally responsive approaches that address structural determinants of health. Despite growing calls for data equity, major U.S. surveillance systems, including the Behavioral Risk Factor Surveillance System (BRFSS) and the National Health Interview Survey (NHIS), rarely collect or report country-of-origin data for African immigrants (Omenka et al. 2020). When such data are available, they are often excluded from public-use files, limiting the ability to identify subgroup disparities or design targeted interventions (Castañeda et al. 2015; Ponce et al. 2025).

This systematic review addresses these gaps by synthesizing evidence on cervical cancer screening and prevention among African immigrant women in the United States. Specifically, we (a) summarize barriers and facilitators to screening, (b) examine how existing studies conceptualize African immigrant identity and employ disaggregated analyses, and (c) apply intersectionality and stress process frameworks to highlight structural determinants shaping screening behaviors. By centering African immigrant women in cervical cancer research, this review aims to advance data equity and inform culturally tailored, evidence-based interventions to reduce preventable disparities.

## Methods

### Search strategy and eligibility criteria

Potential studies were identified through PubMed, ProQuest, EBSCO, PsycINFO, MEDLINE, Scopus, and Google Scholar. Databases were searched for studies conducted in the United States and published in English between January 2010 and December 2024, in accordance with PRISMA 2020 guidelines (Page et al. 2021). The review protocol was registered with PROSPERO (CRD420251151600). The study objective and conceptualization of relevant factors were informed by an a priori review of the literature and recognition of limitations in existing cervical cancer research on immigrant populations.

The search strategy combined controlled vocabulary (e.g., MeSH terms) and free-text keywords, with Boolean operators (“*AND*,” “*OR*”*) used to maximize sensitivity and specificity. Search terms captured population, exposure, and contextual dimensions, including: (a) population terms (e.g., “African immigrant women,” “African-born women,” “sub-Saharan African immigrants,” “foreign-born Black women”); (b) exposure terms (e.g., “cervical cancer,” “Pap test,” “HPV vaccination,” “HPV screening”); and (c) contextual terms (e.g., “United States,” “health disparities,” “language barriers,” “structural inequalities”).* The full electronic search strategy for PubMed is provided in Supplementary Appendix A, in accordance with PRISMA 2020 recommendations.

Reference lists of included articles and relevant reviews were hand-searched to identify additional eligible studies. Gray literature sources (e.g., government health reports and organizational publications) were reviewed for background context; however, only peer-reviewed empirical studies were included in the final synthesis.

### Inclusion and exclusion criteria

Studies were eligible for inclusion if they examined cervical cancer screening, prevention, or HPV vaccination among African immigrant women in the United States and reported findings separately for African immigrants or provided sufficient data to distinguish this subgroup from broader racial or immigrant populations. Eligible studies included women aged 18 years or older, consistent with U.S. cervical cancer screening guidelines, and were published in peer-reviewed journals in English between January 2010 and December 2024. Observational, qualitative, mixed-methods, and interventional designs using primary or secondary data were eligible. Additionally, studies were excluded if they focused exclusively on populations outside the United States without disaggregated African immigrant data; aggregated African immigrants into undifferentiated “*Black*” *or* “*foreign-born*” categories; or were commentaries, editorials, conference abstracts, theses, policy briefs, systematic reviews, meta-analyses, or theoretical papers lacking original empirical data.

### Screening and study selection

**Figure 1.**
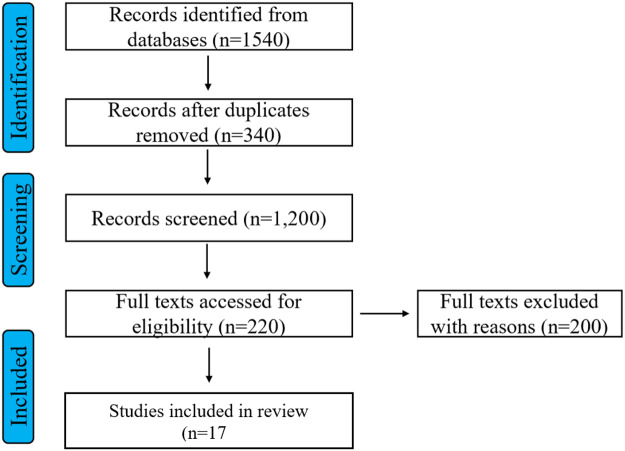
PRISMA flowchart.

All records were imported into reference management software and de-duplicated. Screening proceeded in 2 stages: (1) title and abstract screening and (2) full-text review using predefined inclusion and exclusion criteria. One reviewer (D.B.S.) independently screened all records using a standardized protocol; a second reviewer (S.F.) independently screened a random 20% subset of titles and abstracts to verify reliability and consistency in application of inclusion criteria. Agreement between reviewers was 95%, and all discrepancies were resolved through discussion and consensus. Given the high inter-rater agreement observed in the reliability check, the remaining records were screened by the primary reviewer with regular consultation to ensure consistency. For full-text review, both reviewers independently assessed all articles for final inclusion, with disagreements resolved through consensus discussion. The study selection process is summarized using a PRISMA 2020 flow diagram (Figure 1).

### Data extraction

Data were extracted into a standardized Excel spreadsheet capturing study characteristics (author, year, location, design, sample size), population descriptors, screening and vaccination outcomes, barriers and facilitators, and recommendations. The use of theoretical frameworks, including intersectionality and the stress process model, was recorded, as was whether analyses were disaggregated by country of origin, language proficiency, or immigration characteristics. Two reviewers independently conducted data extraction, with discrepancies resolved by consensus.

### Risk of bias assessment

Methodological quality was assessed using design-appropriate tools. Observational studies were evaluated using the Newcastle–Ottawa Scale (NOS), which assesses selection, comparability, and outcome domains (Wells et al. 2011). Qualitative studies were appraised using the Critical Appraisal Skills Programme (CASP) checklist. Studies were categorized as low, moderate, or high risk of bias based on established criteria.

### Data synthesis

Given substantial heterogeneity in study designs, population definitions, and outcome measures, findings were synthesized narratively rather than through quantitative meta-analysis. Specific sources of heterogeneity included: (a) variation in screening outcome definitions (e.g., ever screened vs. guideline-adherent screening, self-reported vs. medical record-verified), (b) inconsistent operationalization of African immigrant identity (e.g., country of origin vs. regional aggregation, varying inclusion of Caribbean immigrants), (c) diverse analytic approaches ranging from qualitative exploration to secondary analysis of national datasets, and (d) differences in covariate adjustment and comparison groups across quantitative studies. These methodological differences precluded meaningful statistical pooling while supporting thematic synthesis across structural, cultural, and healthcare system domains. Results were grouped thematically across structural barriers, cultural factors, linguistic and educational barriers, and data system limitations. Quantitative findings were summarized descriptively in tables, while qualitative findings were synthesized thematically. Intersectionality and stress process frameworks were used to contextualize how overlapping structural determinants shape cervical cancer screening disparities among African immigrant women.

## Results

### Study selection

As shown in the PRISMA 2020 flow diagram (Figure 1), a total of 1,540 records were identified across 7 electronic databases. After removal of 340 duplicates, 1,200 records were screened by title and abstract, of which 980 were excluded. The remaining 220 articles underwent full-text review, and 203 were excluded due to lack of disaggregated data on African immigrants, focus on populations outside the United States, absence of original empirical data, or ineligible publication type (e.g., conference abstracts, theses, or reviews). Ultimately, 17 peer-reviewed empirical studies, published between 2013 and 2025, met all inclusion criteria and were included in the final synthesis.

Included studies were: Ndukwe et al. (2013); Morrison et al. (2013); Sewali et al. (2015); Adegboyega et al. (2017); Adegboyega et al. (2021); Adekunle et al. (2021); Adegboyega et al. (2022a, 2022b); Amboree et al. (2020); Cofie et al. (2022), (2024); Adebola et al. (2023); Cudjoe et al. (2020); Pratt et al. (2024); Gyan et al. (2025); Spencer et al. (2023); and Adegboyega et al. (2025).

### Study characteristics and methods

Table 1 summarizes the characteristics of the 17 included studies. Research designs included cross-sectional surveys, qualitative interviews and focus groups, mixed-methods studies, retrospective cohort analyses using clinical or national datasets, and 1 randomized controlled trial. Most studies were conducted in U.S. urban areas with sizable African immigrant populations, including Washington, D.C., Iowa, Kentucky, Minnesota, and Massachusetts. National datasets, such as the NHIS, were analyzed in 2 studies, while community-based recruitment predominated in qualitative research. This methodological diversity enabled examination of both population-level screening patterns and contextual factors shaping cervical cancer prevention among African immigrant women.

**Table 1.** Study characteristics and methods (U.S. context; African immigrant or foreign-born Black subgroups)

Study (author, year)	Population & setting	Design/data source	Primary outcome
Ndukwe et al. 2013	African-born women, Washington DC area	Cross-sectional survey (community)	Knowledge and uptake of Pap testing and mammography.
Morrison et al. 2013	Somali women in a U.S. primary care system	Retrospective EHR cohort (2-year window)	Adherence to cervical cancer screening guidelines.
Sewali et al. 2015	Somali immigrants, U.S. Midwest	Community RCT: clinic Pap vs. HPV self-sampling	Screening completion among under-screened women.
Adegboyega et al. 2017	African immigrant women, U.S. South/Midwest	Qualitative descriptive	Factors influencing Pap screening use.
Adegboyega et al. 2021	U.S.-born Black & sub-Saharan African immigrant women	Qualitative focus groups/interviews	Values, beliefs, willingness re: Pap & HPV self-sampling.
Adekunle et al. 2021	African immigrant women, Iowa City	Qualitative (FGDs + interviews)	Awareness, Pap uptake barriers, HPV vaccine acceptance.
Adegboyega et al. 2022a	Sub-Saharan African immigrant women, Kentucky	Cross-sectional survey	Social support and Pap screening uptake.
Adegboyega et al. 2022b	African American & sub-Saharan African immigrants, KY	Cross-sectional survey	Beliefs associated with cancer screening behaviors.
Amboree et al. 2020	Foreign-born Black & Latinx adults, U.S.	Cross-sectional (multi-site)	HPV vaccine knowledge and uptake correlates.
Cofie et al. 2022	U.S.- vs. foreign-born Black adults (NHIS 2013–2017)	Secondary analysis, NHIS	HPV vaccination initiation by nativity/region of birth.
Adebola et al. 2023/2024	U.S.-born vs. sub-Saharan African-born Black women	Qualitative	Pap screening experiences and challenges.
Cudjoe et al. 2020	African immigrant women, U.S.	Cross-sectional survey	Prevalence and correlates of Pap testing.
Cofie et al. 2024	Immigrants of African descent, Southeastern U.S.	Mixed methods	Personal networks and Pap screening.
Pratt et al. 2024	Somali patients within U.S. primary care	Implementation research	Integration of HPV self-collect to boost screening.
Gyan et al. 2025	Black immigrant women (African/Caribbean), MA	Qualitative	Screening beliefs and practices among Black immigrants.
Spencer et al. 2023	Large U.S. health system, multi-ethnic	Retrospective cohort	Racial/ethnic disparities in screening pathways; context for subgroup gaps.
Adegboyega et al. 2025	African American & SAI women, U.S.	Community intervention pilot	HPV self-sampling promotion among Black/SAI women.

### Screening outcomes and disparities

Table 2 summarizes screening outcomes and disparities across the included studies. Across quantitative analyses, African immigrant women consistently exhibited lower rates of Pap testing and HPV vaccination compared with U.S.-born Black women or other immigrant groups (Ndukwe et al. 2013; Amboree et al. 2020; Cofie et al. 2022). Reported Pap testing prevalence or guideline adherence ranged approximately from 51% to 66%, with lower uptake consistently observed among women lacking health insurance, with limited English proficiency, or with shorter duration of U.S. residence (Morrison et al. 2013; Cudjoe et al. 2020; Adegboyega et al. 2022a, 2022b). Where reported, HPV vaccination initiation was substantially lower among foreign-born Black women than among U.S.-born Black women, with notable variation by nativity and region of birth (Amboree et al. 2020; Cofie et al. 2022).

**Table 2.** Outcomes and key findings: disparities, barriers/facilitators, actionable levers

Study	Disaggregation/subgroup handling	Core findings & notes
Ndukwe et al. 2013	African-born women analyzed separately	Low knowledge and suboptimal Pap uptake; cultural beliefs and system navigation issues highlighted.
Morrison et al. 2013	Somali vs. non-Somali	Only 51% adherent; greater overall system use predicted adherence.
Sewali et al. 2015	Somali, under-screened	HPV self-sampling increased completion vs. clinic Pap among recent immigrants.
Adegboyega et al. 2017	African immigrants (country-mix)	Provider recommendation, insurance, and trust facilitated screening; stigma and modesty were barriers.
Adegboyega et al. 2021	U.S.-born Black vs. SAI	Willingness to use self-sampling grew with culturally tailored education.
Adekunle et al. 2021	African immigrants (Iowa)	Low baseline awareness; language and logistics key obstacles; strong openness to vaccinating children after explanation.
Adegboyega et al. 2022a	SAI women only	Pap uptake 65.7%; affectionate/positive support associated with screening.
Adegboyega et al. 2022b	SAI vs. African American	Insurance and education predicted screening; fatalism associated with lower uptake in SAI subgroup.
Amboree et al. 2020	Foreign-born Black vs. U.S.-born	Lower HPV vaccine knowledge among foreign-born; structural access gaps.
Cofie et al. 2022	U.S.- vs. foreign-born Black	Foreign-born women had lower HPV vaccine initiation; region-of-birth differences observed.
Adebola 2023/24	U.S.-born vs. SAI	Distinct experiential barriers for SAI, including modesty, language, and system trust; supports subgroup tailoring.
Cudjoe et al. 2020	African immigrants only	Identified correlates of Pap testing including insurance, length of stay, and access.
Cofie et al. 2024	Black immigrants of African descent	Personal network connectedness linked with screening, attenuated after adjustment.
Gyan et al. 2025	Black immigrants incl. African	Culturally tailored resources and community partnerships recommended to promote screening.
Spencer et al. 2023	System-level U.S. dataset	Black patients more likely to receive Pap-only testing and experience follow-up gaps, underlining equity issues relevant to immigrant subgroups.
Adegboyega 2025	Black & SAI women	Community promotion of HPV self-sampling is acceptable and can boost intent/uptake.

Qualitative and mixed-methods studies documented cultural, religious, and structural factors shaping screening behaviors. Beliefs linking cervical cancer to sexual promiscuity or moral transgression contributed to stigma and delayed screening (Adekunle et al. 2021; Gyan et al. 2025). Language barriers, limited familiarity with U.S. healthcare systems, and difficulties navigating insurance and appointment processes were frequently cited obstacles (Adegboyega et al. 2017; Adebola et al., 2023). In contrast, physician recommendation, culturally tailored education, and trusted community-based messaging emerged as consistent facilitators of screening engagement (Sewali et al. 2015; Adegboyega et al. 2021).

Intervention studies, though limited in number, suggested that alternative screening approaches may help reduce access barriers. In a randomized trial, Sewali et al. (2015) demonstrated that HPV self-sampling significantly increased screening completion among under-screened Somali immigrant women compared with clinic-based Pap testing. Implementation-focused studies further indicated that integrating self-collection options into primary care settings serving African immigrant communities is feasible and may improve reach among women facing privacy, modesty, or logistical constraints (Pratt et al. 2024; Adegboyega et al. 2025). Collectively, these findings indicate that interventions addressing both structural access barriers and culturally mediated concerns hold promise for improving cervical cancer prevention among African immigrant women.

### Disaggregation, measurement, and use of theoretical frameworks

As shown in Table 3, 11 of the 17 included studies disaggregated African immigrant women by country of origin or specific national subgroups. The remaining studies aggregated participants under broader categories such as “foreign-born Black” or “African immigrant” without further stratification (Cofie et al. 2022; Spencer et al. 2023). This limited disaggregation restricts the ability to identify high-risk subgroups, particularly given substantial heterogeneity across African nationalities in migration pathways, cultural norms, health beliefs, and patterns of healthcare utilization (Adebola et al., 2023; Gyan et al. 2025). Studies that employed country-level disaggregation were more likely to identify distinct barriers related to modesty norms, prior healthcare experiences, and language-specific communication challenges, whereas aggregated analyses tended to mask these differences.

**Table 3.** Measurement approaches, disaggregation, and theoretical frameworks in included studies (*n* = 17)

Study (author, year)	Immigrant subgroup definition	Country-of-origin data?	Language/LEP measured?	Years in U.S./acculturation measured?	Insurance/access variables?	Theory applied (e.g., intersectionality, stress process)
Ndukwe et al. 2013	African-born women self-identified	Yes – Nigerian, Ghanaian, Ethiopian	Yes – English proficiency noted	Yes	Yes	No explicit theory
Morrison et al. 2013	Somali ethnicity via EHR	Yes – Somali only	No	Yes	Yes	No
Sewali et al. 2015	Somali immigrant status (self-report)	Yes – Somali only	No	Yes	Yes	Health behavior framework
Adegboyega et al. 2017	African immigrants, self-reported country	Yes – Nigeria, Ghana, others	Yes	Yes	Yes	Stress process model
Adegboyega et al. 2021	SAI vs. U.S.-born Black, self-reported	Yes – multiple African nations	Yes	Yes	Yes	Intersectionality + stress process
Adekunle et al. 2021	African immigrants, self-identified	Yes – Nigerian, Sudanese, others	Yes	Yes	Yes	Intersectionality lens
Adegboyega et al. 2022a	SAI only, self-reported	Yes – multiple African nations	Yes	Yes	Yes	Stress process model
Adegboyega et al. 2022b	SAI vs. U.S.-born Black	Yes – stratified	Yes	Yes	Yes	Health belief model
Amboree et al. 2020	Foreign-born Black vs. U.S.-born Black	Region only, not specific country	No	No	Yes	No explicit theory
Cofie et al. 2022	U.S.- vs. foreign-born Black adults	Region-level, not specific country	No	No	Yes	No
Adebola et al. 2023/2024	U.S.-born vs. African-born	Yes – Nigerian, Ghanaian, others	Yes	Yes	Yes	Intersectionality lens
Cudjoe et al. 2020	African immigrants, self-reported	Yes – multiple African nations	No	Yes	Yes	No explicit theory
Cofie et al. 2024	African descent immigrants	Region only	No	Yes	Yes	Social network theory
Pratt et al. 2024	Somali only, clinical sample	Yes – Somali	No	Yes	Yes	Implementation science
Gyan et al. 2025	Black immigrants (African & Caribbean)	Region-level only	No	No	Yes	Intersectionality
Spencer et al. 2023	Multiethnic system data	Race/ethnicity only, no immigrant flag	No	No	Yes	No
Adegboyega et al. 2025	African American & SAI women	Yes – SAI identified	Yes	Yes	Yes	Intersectionality + stress process

Measures of language proficiency were included in 10 studies, most commonly operationalized through self-reported English proficiency or preferred language of healthcare communication. Across these studies, limited English proficiency was consistently associated with lower cervical cancer screening uptake and greater difficulty navigating preventive services (Adegboyega et al. 2017; Amboree et al. 2020). However, few studies examined language access beyond individual proficiency, such as availability of interpreter services or language concordance with providers.

Indicators of acculturation or migration exposure were more commonly assessed. Fourteen studies measured years of residence in the United States or related migration characteristics. Longer duration of U.S. residence was generally associated with higher screening adherence, greater awareness of HPV and cervical cancer prevention, and increased engagement with routine healthcare (Morrison et al. 2013; Ndukwe et al. 2013). Nonetheless, the operationalization of acculturation varied widely, and few studies examined how length of residence interacted with structural factors such as insurance eligibility or legal status.

The application of theoretical frameworks was uneven across the evidence base. Only 7 studies explicitly applied conceptual frameworks to interpret screening disparities among African immigrant women. Intersectionality theory and the stress process model were the most frequently used approaches, often to situate screening behaviors within overlapping systems of racialization, gendered expectations, immigration-related stressors, and resource constraints (Adegboyega et al. 2021; Adebola et al. 2023; Gyan et al. 2025). Earlier studies were largely descriptive and lacked explicit conceptual grounding, while more recent research increasingly incorporated theory to contextualize individual behaviors within broader structural and social processes.

## Discussion

To the best of our knowledge, this is the first systematic review to synthesize evidence on disparities in cervical cancer screening and prevention among African immigrant women in the United States. Drawing on 17 peer reviewed empirical studies, this review demonstrates that African immigrant women experience persistent gaps in Pap testing and HPV vaccination that are shaped by intersecting structural, cultural, and healthcare system factors. Although the included studies varied in data sources, sampling strategies, and analytic approaches, they consistently documented lower screening uptake among African immigrant women compared to U.S. born Black women and other immigrant groups (Amboree et al. 2020; Adegboyega et al. 2022b; Cofie et al. 2022).

A central challenge across the literature is inconsistency in how African immigrant populations are defined and analyzed. Some studies disaggregated African immigrants by country of origin or specific national subgroups, enabling identification of distinct cultural norms, language needs, and prior healthcare experiences that shaped screening behaviors (Ndukwe et al. 2013; Adebola et al., 2023). In contrast, other studies aggregated participants into broad categories such as “foreign born Black” or “African immigrant,” which limited the ability to identify within group heterogeneity and high risk subpopulations (Cofie et al. 2022; Spencer et al. 2023). This lack of standardization complicates cross study comparison and constrains conclusions about subgroup specific disparities.

Despite these methodological differences, several barriers to cervical cancer screening emerged consistently across studies. Language discordance, lack of health insurance, unfamiliarity with the U.S. healthcare system, cultural stigma surrounding gynecological examinations, and limited awareness of HPV and cervical cancer prevention were repeatedly identified as impediments to screening (Adekunle et al. 2021; Adebola et al. 2023; Gyan et al. 2025). Screening uptake was particularly low among women with limited English proficiency, shorter duration of residence in the United States, and no regular source of care (Morrison et al. 2013; Adegboyega et al. 2017). These patterns point to the combined influence of individual level barriers and broader structural constraints on preventive care access.

A smaller subset of studies examined intervention strategies to address these gaps. Community-based education initiatives and HPV self-sampling interventions were associated with improved screening engagement among African immigrant women, particularly when designed to address concerns related to privacy, modesty, and trust in healthcare systems (Sewali et al. 2015; Adegboyega et al. 2025). These findings suggest that interventions tailored to the lived experiences of African immigrant women may be more effective than standard clinic-based approaches.

Theoretical frameworks were applied unevenly across the literature. Only a limited number of studies explicitly used intersectionality theory or the stress process model to interpret screening disparities (Adegboyega et al. 2021; Adebola et al. 2023; Gyan et al. 2025). Studies that employed these frameworks were better positioned to situate screening behaviors within overlapping systems of racialization, gendered expectations, immigration related stressors, and constrained resources. Intersectionality highlights how multiple forms of marginalization operate simultaneously, while the stress process model emphasizes the role of chronic stressors and limited coping resources in shaping health behaviors (Pearlin et al. 1981; Crenshaw 1991). The limited application of these frameworks across studies represents a missed opportunity to move beyond descriptive accounts toward more explanatory analyses of cervical cancer screening disparities.

Essentially, the findings indicate that disparities in cervical cancer prevention among African immigrant women are not solely the result of individual knowledge deficits or cultural beliefs but reflect broader structural conditions related to healthcare access, language, immigration, and social positioning. Strengthening data disaggregation, expanding theoretically informed research, and supporting culturally responsive screening approaches are critical steps toward addressing these persistent inequities.

These prevention disparities have direct implications for palliative and supportive care. Delayed or forgone cervical cancer screening among African immigrant women contributes to later-stage diagnoses, when disease has progressed beyond curative treatment and requires intensive symptom management, psychosocial support, and end-of-life care (American Cancer Society 2025). Late-stage cervical cancer presents with complex symptom burdens including pain, bleeding, urinary and bowel dysfunction, and significant psychosocial distress, necessitating coordinated palliative interventions (World Health Organization 2020). For African immigrant women already facing language barriers, cultural distance from healthcare systems, and limited social support networks, late presentation compounds the challenges of accessing appropriate palliative services (Omenka et al. 2020). Furthermore, cultural beliefs surrounding illness disclosure, family decision-making, and end-of-life preferences may not align with Western palliative care models, underscoring the need for culturally responsive supportive care approaches (Gyan et al. 2025). By addressing screening barriers upstream, we can reduce the burden of advanced disease and the subsequent demand for complex palliative services, while simultaneously ensuring that supportive care systems are prepared to serve African immigrant women with cultural humility and linguistic accessibility when prevention fails

## Limitations

This review has several limitations that should be considered when interpreting the findings. The evidence base focused on African immigrant women in the United States remains relatively small and methodologically heterogeneous, with many studies relying on small convenience samples or qualitative designs that limit generalizability. Although this review followed PRISMA guidelines and included comprehensive searches of multiple databases, Google Scholar, and reference lists, it is possible that relevant gray literature and community-based reports were not captured.

The substantial diversity of African immigrant communities by country of origin, language, migration history, and time in the United States further constrains transferability. Findings from 1 national or regional subgroup may not apply to others, even within the broader category of African immigrant women. In addition, variation in how studies defined and operationalized immigrant status, screening outcomes, and comparison groups limited cross study comparability.

Measurement limitations in the primary studies also warrant consideration. Many quantitative studies relied on self-reported screening behaviors, which may be subject to recall bias or misclassification, particularly among recent immigrants with varying understandings of Pap testing and HPV vaccination. Finally, because this review employed a narrative synthesis rather than quantitative pooling, thematic prioritization and interpretation involved a degree of analytic judgment.

Despite these limitations, this review consolidates fragmented evidence, highlights critical gaps in data disaggregation and theory use, and identifies actionable directions for future research, policy, and practice aimed at reducing cervical cancer screening disparities among African immigrant women in the United States.

## Conclusion

African immigrant women in the United States experience clear and preventable gaps in cervical cancer prevention, and closing these gaps requires coordinated action that begins with visibility and ends with delivery. Evidence from this review directly supports several recommendations. First, disaggregated measurement is essential; studies that stratified by country of origin or national subgroup (*n* = 11) were better able to identify distinct barriers related to language, cultural norms, and prior healthcare experiences than those using aggregated categories (Ndukwe et al. 2013; Adebola et al. 2023). Health agencies, cancer registries, and electronic health record systems should routinely capture and publish country-of-birth data to make subgroup disparities visible and progress trackable. Second, evidence from intervention studies demonstrates that HPV self-sampling and culturally tailored education significantly improve screening completion among under-screened African immigrant women (Sewali et al. 2015; Adegboyega et al. 2025), supporting broader implementation of these approaches. Additional policy recommendations, while not directly evaluated in the reviewed studies, are justified by documented structural barriers and equity considerations. These include expanding Medicaid in remaining states, removing or waiving the 5-year waiting period for preventive services, extending publicly funded primary and preventive care regardless of immigration status, and clearly communicating confidentiality protections so fear does not deter care seeking – all of which address insurance gaps, unfamiliarity with U.S. healthcare systems, and immigration-related concerns consistently identified across studies.

Evidence-based health system strategies include investing in interpreter services and language-concordant care, as language barriers were consistently associated with lower screening uptake across multiple studies (Adegboyega et al. 2017; Amboree et al. 2020); implementing community health worker and patient navigation programs, which align with documented needs for healthcare system guidance and trusted intermediaries (Cofie et al. 2024); and delivering services in community settings such as faith centers and cultural organizations, where qualitative research indicates African immigrant women feel more comfortable accessing care (Adekunle et al. 2021; Gyan et al. 2025). Complementary strategies supported by equity principles but requiring further evaluation include culturally responsive provider training and workforce diversification, mobile screening initiatives, co-creation of public health outreach with community and faith leaders, and school-based HPV vaccination programs tailored to address parental concerns documented in qualitative studies. While these approaches are theoretically grounded and ethically justified, rigorous implementation research is needed to determine their effectiveness and optimal delivery models in African immigrant communities.

Future research can reinforce these efforts by using mixed methods approaches to quantify subgroup specific disparities and to explain mechanisms within particular national origin groups. Greater application of implementation science is needed to test which combinations of patient navigation, language support, insurance coverage, and clinic workflow adaptations most effectively increase screening and vaccination uptake. The use of shared, disaggregated indicators across studies would also facilitate comparison and enable evaluation of progress over time.

Cervical cancer is largely preventable. Ensuring that African immigrant women are visible in data, prioritized in policy, and served by culturally grounded programs represents both sound public health practice and a matter of equity. Such efforts are essential for closing persistent gaps in prevention and for advancing national goals in cervical cancer control.

## Supporting information

10.1017/S1478951526101849.sm001 Suleman and Folorunsho supplementary material Suleman and Folorunsho supplementary material

## Supplementary Material

The supplementary material for this article can be found at https://doi.org/10.1017/S1478951526101849.
