# Supplementary material for: Cervical cancer research disparities among African immigrant women in the United States: A systematic review
Source: Palliat Support Care. 2026 Feb 24;24:e68. doi: 10.1017/S1478951526101849 (PMC13166448; doi:10.1017/S1478951526101849)
Supplement: Suleman and Folorunsho supplementary material [file S1478951526101849sup001.docx]

**Supplementary Appendix A**

**Supplementary Table A1. Full Electronic Search Strategy for PubMed**

| **Search Component** | **Search Terms / Syntax** |
| --- | --- |
| **Population (African immigrant women)** | “African immigrant women”[Title/Abstract] OR “African immigrants”[Title/Abstract] OR “African-born women”[Title/Abstract] OR “sub-Saharan African immigrants”[Title/Abstract] OR “foreign-born Black women”[Title/Abstract] OR (“Emigrants and Immigrants”[MeSH] AND Africa[MeSH]) |
| **Condition / Outcome (Cervical cancer & prevention)** | “Cervical Cancer”[MeSH] OR “Uterine Cervical Neoplasms”[MeSH] OR “cervical cancer screening”[Title/Abstract] OR “Pap test”[Title/Abstract] OR “Pap smear”[Title/Abstract] OR “HPV vaccination”[Title/Abstract] OR “HPV screening”[Title/Abstract] OR “human papillomavirus”[Title/Abstract] |
| **Context (United States)** | “United States”[MeSH] OR “United States”[Title/Abstract] OR U.S.[Title/Abstract] |
| **Combined Boolean Logic** | (Population) AND (Condition/Outcome) AND (Context) |
| **Limits Applied** | English language; Human studies; Publication dates January 1, 2010 – December 31, 2024 |
| **Database** | PubMed |
| **Search Date** | December 2024 |

**Supplementary Notes**

1. The PubMed search strategy combined MeSH terms and free-text keywords to maximize sensitivity.
2. Retrieved records were exported to reference management software for de-duplication.
3. Results were screened alongside records from ProQuest, EBSCO, PsycINFO, MEDLINE, Scopus, and Google Scholar.
4. Grey literature (e.g., CDC and ACS reports) was reviewed for contextual background only and was not included in the final analytic sample.
